# Supplementary material for: Investigating the role of AI explanations in lay individuals’ comprehension of radiology reports: A metacognition lens
Source: PLoS One. 2025 Sep 10;20(9):e0321342. doi: 10.1371/journal.pone.0321342 (PMC12422517; doi:10.1371/journal.pone.0321342)
Supplement: Attachment — Submitted filename: Response to Reviewers.docx [file pone.0321342.s001.docx]

**Current Manuscript Title:** Investigating the Role of AI Explanations in Lay Individuals’ Comprehension of Radiology Reports: A Metacognition Lens

**Summary of Changes to the Manuscript**

We are tremendously grateful to you and the review panel for seeing value in this paper, the careful reading of the manuscript, the constructive and well-thought feedback provided, and the opportunity to revise our work. Based on the recommendations of the review team, we substantially updated the literature review, clarified the methods for model building and statistical analysis, and sharpened our findings and limitations.

In addition to these significant changes, we have addressed or responded to all other issues raised by the review panel. Our point-by-point responses are elaborated next. We hope you find that this revision addresses the core concerns that were raised. Thank you again for the opportunity to revise the manuscript. We look forward to any feedback you may have about how to improve this work further.

**Response to Reviewer 1 Comments**

|  | **Reviewer 1’s Comment** | **Authors’ Response** |
| --- | --- | --- |
| R1.1 | The study is technically sound. The hypotheses are clearly stated and rooted in prior theory. The random assignment to explanation formats, the use of real chest X-rays, and the employment of both behavioral and self-reported outcome measures collectively support strong internal validity. The conclusions are supported by appropriate statistical analysis using logistic regression and stratified comparisons. | We sincerely thank you for the merit you see in our work and are grateful for the opportunity to revise our work. |
| R1.2 | The manuscript is also notable for its transparency. The dataset, experimental materials, and code are made fully available, adhering to open science best practices. This enhances the credibility and reproducibility of the study. |  |
| R1.3 | The writing is clear, and the results are communicated in an accessible way. The framing using metacognitive "direct access" versus "cue-based inference" provides a novel lens on the efficacy of explanations in XAI, and this framing is well-motivated throughout. |  |
| R1.4 | Consider discussing the potential influence of participants' prior knowledge or medical familiarity on their comprehension. | We added this discussion to the fourth paragraph in Section 5.2. The added context for your reference is as below:  Fourth, another important consideration is the role of participants’ prior medical knowledge in shaping their comprehension of both radiology report content and AI-generated explanations. Although we included a self-reported measure of healthcare literacy to approximate domain expertise, this measure provides only a general sense of participants' familiarity with medical concepts. Individuals with more extensive medical knowledge may interpret technical terminology or explanation cues differently, potentially moderating the impact of AI support on decision outcomes. Conversely, those with less familiarity may benefit more from explanatory cues that simplify or contextualize medical language. Future research should explore how varying levels of domain-specific knowledge influence human-AI collaboration, particularly in high-stakes domains like healthcare, and consider incorporating more granular measures of expertise to better tailor AI explanations to user needs. |
| R1.5 | It would be valuable to elaborate briefly on how the findings might generalize to other modalities or clinical contexts beyond chest X-rays. | We have added a paragraph in the Discussion section (the fifth paragraph of Section 5.2) outlining how the underlying mechanisms—particularly metacognitive self-monitoring influenced by AI explanations—may extend to other clinical contexts, such as pathology reports or diagnostic summaries in other specialties. The added context for your reference is as below:  Fifth, although our study focused on radiology report excerpts related to anatomical assessments, the mechanisms uncovered—particularly how AI explanations influence self-monitoring and final decision accuracy—may generalize to other clinical contexts. In domains such as pathology, dermatology, or even mental health assessments, clinicians and patients routinely interpret textual or semi-structured diagnostic data. In these settings, explanatory cues that highlight salient features or align with human reasoning may similarly support cognitive calibration and improve judgment. Future studies should explore the transferability of these effects across modalities and specialties to assess how explanation design can be adapted to domain-specific workflows and reasoning styles. |
| R1.6 | A short reflection on the limitations of using synthetic AI explanations (rather than explanations from deployed systems like ChatGPT or GlassBox models) might help ground expectations for real-world translation. | Thanks for bringing this we now added the following to the end of section 5.3  While our study used feature-based explanations generated from an SVM trained on radiology report sentences, it is important to recognize that these explanations are synthetic in nature. That is, they were created specifically for this experiment using controlled, transparent mechanisms (e.g., ranked unigrams and bigrams). In contrast, real-world AI systems such as ChatGPT or GlassBox models often produce explanations that are generated through more complex, probabilistic, and potentially less transparent methods. These real-world systems may involve dialogue-based, multi-modal, or dynamically updated explanations shaped by conversational context. As such, the effects of explanations observed in our study may not fully generalize to real-world deployments where explanations are noisier, more ambiguous, or inconsistently interpreted. Future research should explore whether the self-monitoring benefits observed here persist under more realistic, end-to-end AI explanation interfaces. |

**Response to Reviewer 2 Comments**

|  | **Reviewer 2’s Comment** | **Authors’ Response** |
| --- | --- | --- |
| R2.1 | The paper is well-structured and presents some interesting findings, but there are several significant issues that need to be addressed before publication. | Thank you for carefully considering our manuscript. We have significantly revised our manuscript and have addressed all the issues raised. We also thank you again for your constructive feedback and hope that you find the revised manuscript satisfactory. |
| R2.2 | The most critical oversight is the absence of any discussion on "reliance", which is a fundamental concept in the Human-Computer AI Interaction literature. This paper clearly investigates how humans rely on AI with added explanations, yet fails to position itself within this important body of literature. Established measures such as Relative Self-Reliance and Relative AI Reliance could have been adopted to strengthen the analysis. | Thank your pointing us the growing literature on reliance in the Human-AI interaction domain. While our primary framing hypotheses centered on participants’ initial framing, we agree that reliance on AI recommendations without careful consideration (e.g. due to behavioral limitations and systematic biases) is an important factor in AI-augmented assisted decision making.  We extended our limitation and future research (Section 5.3) by addressing this limitation. Specifically, we added the following:  Finally, our study primarily focuses on participants’ initial framing, and their deliberate consideration of AI recommendations when presented. While we control for participants’ trust in our work, there is a growing emphasis on the importance of calibrating trust (i.e., avoiding over- or under-reliance [14]) to enhance AI-assisted decision-making [92]. We expect further studies to consider the nuanced differences between trust and reliance [16,29] in studying the effects of explanations on metacognitive processes of AI-assisted decision-making. |
| R2.3 | The choice of participants raises serious concerns about ecological validity. Using a general audience from Mechanical Turk to perform specialized medical diagnosis tasks is problematic. The authors should justify why they didn't use participants with relevant expertise or at least address this limitation more thoroughly. | We fully acknowledge that lay individuals do not represent expert clinicians and that this may limit the direct applicability of our findings to real-world clinical workflows. However, our primary objective was to examine how AI explanations affect decision-making processes, particularly self-monitoring mechanisms, in individuals without domain expertise—such as patients or general healthcare consumers—who increasingly interact with AI-enabled tools for interpreting test results and medical information. This aligns with growing efforts to design AI systems not only for clinicians but also for end-users seeking to understand their own health data. We have now expanded the discussion of this limitation and clarified our rationale for using non-expert participants in the revised manuscript. Accordingly we added the following discussion to Section 5.3. of the revised manuscript.  Additionally, this study used lay participants recruited from Amazon Mechanical Turk rather than individuals with formal medical training. While this choice may constrain ecological validity for clinical decision-making settings, it reflects a deliberate focus on understanding how general users (e.g., lay patients) interpret AI explanations in the absence of domain expertise. As AI-based tools become increasingly available to patients and the broader public—for example, in direct-to-consumer health platforms or patient-facing EHR systems—understanding how lay individuals rely on AI and engage in self-monitoring is crucial. |
| R2.4 | The proxy measures used to categorize participants are questionable. Self-reported healthcare literacy is an overly broad measure that may not accurately reflect domain expertise for the specific task of interpreting radiology reports. Similarly, self-reported has well-documented limitations in the literature that aren't adequately addressed. | Thank you for pointing this out. The issue has also been raised by another reviewer (in R3.2.) We addressed this point in our limitations section by adding the following paragraph  We would like to note that we measured domain expertise using a self-reported healthcare literacy item, which served as a proxy for familiarity with medical terminology and context. However, we acknowledge that this is a coarse measure that does not account for broader forms of domain-general expertise. |
| R2.5 | The methodology for creating models with different accuracy levels is poorly explained and justified. The authors trained different models on different training set sizes to simulate varying accuracies, but they don't report the actual accuracy differences between these models. Moreover, using differently sized training sets may result in models that are differently fitted rather than having meaningfully different accuracies. | We appreciate your thoughtful comment and agree that additional clarification on our methodology is warranted. To address this, we have revised the manuscript to better describe the creation and evaluation of our “high-accuracy” and “low-accuracy” models. We added the following to section 3.2.2 of the paper:  In particular, we created two models: a “high-accuracy model” trained on the full set of 727 samples, and a “low-accuracy model” trained on a randomly selected 20% subset of the same data. The performance of the two models was evaluated using the AUC metric on the experimental dataset containing 276 expert-labeled sentences. In addition, we report accuracy, calculated as the proportion of correct predictions:  Accuracy= (Number of Correct Predictions)/(Total number of prediction).  Based on this measure, the “low-accuracy model” achieved an accuracy of 0.702, while the “high-accuracy model” achieved a substantially higher AUC of 0.891, confirming the intended disparity in predictive performance. To assess the statistical significance of this difference, we conducted DeLong’s test for two correlated ROC curves, which yielded a p-value of 1.83 ×10^(-10) , indicating a highly significant difference between the two models.  We hope that you find our response satisfactory. |
| R2.6 | "Knowledge of AI technology" is likely not a good indicator of algorithmic literacy, and this limitation is not acknowledged. | Thank you for this insightful observation. We agree that self-reported knowledge of AI technology may not fully capture participants' algorithmic literacy, which involves a more nuanced understanding of how algorithms operate, their limitations, and appropriate contexts for their use. Our intent was to approximate familiarity with AI tools as a control variable, but we acknowledge that it is an imperfect measure of true algorithmic literacy. We have now added a note in the Limitations section to explicitly recognize this distinction and recommend that future work incorporate more validated or performance-based measures of algorithmic literacy. For your convenience the added content is presented below:  Similarly, while we included a self-reported measure of participants’ knowledge of AI technology to approximate their familiarity with algorithmic systems, we acknowledge that this is not an ideal proxy for algorithmic literacy. Algorithmic literacy entails an understanding of how AI systems generate predictions, their underlying assumptions, and appropriate interpretations of their outputs. Our measure likely reflects general familiarity or comfort with AI concepts rather than this deeper cognitive skill set. Future studies should consider incorporate more rigorous assessments of participants’ medical familiarity and algorithmic literacy to better capture how these factors shape human-AI collaboration. |
| R2.7 | The statistical tests used in the analysis lack justification, and the reporting of significance is incomplete. While the authors claim their hypotheses are supported, they should clarify whether all supported hypotheses have p-values < 0.05. Additionally, the paper would benefit from more detailed reporting of effect sizes to help readers understand the practical significance of the findings. | We appreciate your note on this. We initially discussed our analytical tests (4.1., 4.2 and 4.3) and report on their significance under the subsections of each result or highlight them multivariate analysis table. We extended our justifications in these sections. To make it easier for the readers, we added our p-values that were reported in these sections to the summary of findings table (Column: Findings).  We also appreciate your suggestion about reporting on effect sizes and helping the readers understand practical significance of the results. We added a column to Table 5. |
| R2.8 | The literature review is outdated, with only three papers from 2023 and nothing more recent. This is a rapidly evolving field, and the authors should incorporate more current research. | Thank you for pointing this out. We resurveyed the literature to incorporate more recent developments in the field. Particularly, section 2.1 XAI: Explainable AI of the literature review includes more recent studies. Overall, we now have eighteen papers that are from 2023 or more recent. |

**Response to Reviewer 3 Comments**

|  | **Reviewer 2’s Comment** | **Authors’ Response** |
| --- | --- | --- |
| R3.1 | I enjoyed reading this paper, not only because it was executed and written well (S2), but also because it studies an important issue (the interaction between human and AI in joint decision making) and the focus of the study is rather intriguing: meta-cognitive processes (S1). Being an AI expert and not a social scientist, I welcome the aid of social scientists with works like these for the purpose of improving the design of AI-support in decision support systems. | We are grateful for your careful consideration and highlighting the strengths of the manuscript. |
| R3.2 | W1: I wondered about a few things when analysing your research design. First, isn't it the case that people of varying intelligence may have different meta-cognitive processes? I would have expected some aspect(s) associated with intelligence or level of schooling to be gathered from the participants (table 2) and analysed. Second, the participants form a population that is quite different from the population of healthcare professionals. One should be very careful with extrapolating conclusions to decision making by healthcare professionals. Thirdly, you formulated hypotheses around initial framing, but there is another important case of framing: an apriori assumption that the AI will be correct. I have witnessed this phenomenon with healthcare professionals placing overly strong trust in AI recommendations. Fourthly, in section 3.4.5 you discuss the control variables among which "domain expertise". I find this a bit too crude: a professional with expertise in decision making from another domain is very likely to exhibit similar meta-cognitive behavior while in decision making in their own domain or in health. I don't expect that you can fix all the points I raised, nor that all of them are valid, but if you agree with one or more of my comments, then I would welcome them mentioned as a limitation of the study. | We sincerely thank the reviewer for these valuable and thoughtful observations regarding our research design and interpretation. We respond to each of the four points below and have revised the manuscript accordingly to acknowledge relevant limitations and potential future research directions.  **Cognitive Ability and Metacognition:**  We agree that individual differences in cognitive ability, such as general intelligence or educational level, are known to influence metacognitive processes. While we did not explicitly collect formal measures of intelligence or schooling level, we addressed this in part by applying inclusion criteria that required participants to be experienced, English-speaking Mechanical Turk workers with at least 1,000 previously approved HITs and a minimum 95% approval rate. These criteria are commonly used to ensure baseline cognitive competence, attentiveness, and task reliability in online experimental studies. In addition, we collected self-reported measures of participants’ technical literacy and AI literacy, which we included as control variables in our regression analyses to account for individual variation in comfort and familiarity with technology-supported decisions. We also embedded comprehension checks and ensured that the task instructions and interface were designed to be accessible to a general adult population. Nevertheless, we acknowledge this as a limitation and further clarified this aspect in Section 3.3.  **Generalizability to Healthcare Professionals:**  We fully agree with your caution against overgeneralizing from a lay population to professional healthcare decision-makers. While our study provides foundational insights into metacognitive engagement with AI explanations, we have revised the discussion to more clearly note that our findings should not be directly extrapolated to clinical practice without further empirical validation. We also suggest that follow-up research could replicate our experiment with medical trainees or clinicians to better assess ecological validity in healthcare contexts. In particular, we added the following to the limitations.  We would like to note that we measured domain expertise using a self-reported healthcare literacy item, which served as a proxy for familiarity with medical terminology and context. However, we acknowledge that this is a coarse measure that does not account for broader forms of domain-general expertise.  **A Priori Trust in AI:**  We appreciate this important point. While our primary framing hypotheses centered on participants’ initial framing, we agree that an a priori assumption about AI correctness is another critical factor in how individuals engage with AI advice. Although we did not directly manipulate or isolate this type of framing, we included a self-reported measure of trust in AI as a control variable in our regression models to partially account for individual differences in baseline attitudes toward AI systems. We also clarified and incorporate the following sentence in the text:  Moreover, trust also serves as a proxy for participants’ a priori assumptions about the correctness or reliability of AI predictions—a form of baseline framing that may influence how individuals interpret and integrate AI recommendations.  **Granularity of Domain Expertise Measures:**  We appreciate your comment and agree that expertise in other professional fields may influence how individuals engage in complex decisions. However, our study focused on a task requiring familiarity with medical terminology and context, where specific knowledge of healthcare language is likely to affect participants’ ability to interpret both the radiology report and the AI-generated output. For this reason, we used self-reported healthcare literacy to account for differences in task-relevant knowledge. While this measure does not capture broader professional experience or cognitive strategies developed in other fields, it served our purpose of adjusting for familiarity with the specific content. |
| R3.3 | W2: In some ways, working with an AI providing a second opinion is different than working with a human providing a second opinion ... but in other ways, they are not so different. I assume there are studies about effects on decision making when receiving a second opinion from another professional with or without an explanation. I would welcome a short discussion in this paper in what way your results show differences or commonalities between teaming up with an AI or teaming up with a human professional in a similar way. | We agree that comparing human-AI collaboration to human-human second opinion dynamics is an important avenue for understanding the nature of decision support. While our study was not explicitly designed to contrast AI with human collaborators, our findings do offer implications for how users engage with advice depending on the presence and quality of supporting explanations. We have added a short paragraph to the Discussion section (Section 5.2) to reflect on the parallels and distinctions between AI- and human-based second opinions, particularly in terms of perceived expertise, trust calibration, and metacognitive engagement. The new added context is copied below for your convenience:  Sixth, our study also invites comparison to research on second opinions provided by human professionals. Similar to human collaborators, AI systems offering a second opinion can influence decisions through both the content of the recommendation and the presence of a rationale or explanation. However, unlike human experts, AI systems often lack social cues, experiential context, and perceived accountability—all of which can shape how advice is received and evaluated. Prior research in team cognition (e.g., [13,112]) has shown that explanations from human peers can enhance advice acceptance by promoting understanding and credibility. In clinical settings, second opinions are valued not only for diagnostic correction but also for building confidence and reducing decisional conflict [32]. AI explanations, while more mechanical, may similarly scaffold user reasoning by offering interpretable anchors. Our results suggest that explanations from AI can trigger self-monitoring and override intuitive errors, much like a persuasive human second opinion might. However, the path to trust and reliance likely differs due to the perceived agency, intent, and contextual awareness that human collaborators bring. Future research should explore whether users internalize and weigh second opinions from AI systems differently than those from human experts, particularly in high-stakes domains such as healthcare. |
| R3.4 | W3: In Section 3.2.1 you say that you use the expert labels of the 276 sentences as golden standard to evaluate the AI predictions and participants' decision outcomes. Section 3.4.1 describes these accuracy metrics, but I fail to recognize the use of the golden standard in the explanation of the metrics. It only speaks of majority vote. Please clarify this apparent inconsistency. Furthermore, why not provide a simple formula for the metrics? It is much more exact and easier to interpret than a description of a formula in natural language. In Section 3.4.4 you describe how you measure the self-confidence. If I understand correctly, participants are assigned to a group, so a participant in the "with explanations" group will never see sentences under the control condition. So, how do you determine their self-confidence? Furthermore, do I understand correctly that the self-confidence is per candidate and not per sentence (like the AI's confidence)? Please clarify these aspects. | Thank you for pointing these unclarities. Our accuracy evaluations are based on expert responses. We used majority voting to aggregate responses from the independent participants who repeated the same task (under each condition). We clarified this in section 3.4.1  As for the self-confidence, we measured the self-confidence per sentence (i.e. task) since our unit of analysis was tasks. Since the same sentences were used in each condition, we measure the confidence among the three participants under the control condition (since participants did not see any AI prediction in this condition) and calculated the average self-confidence score for the task. We revised section 3.4.4 to clarify this point. |
| R3.5 | W4: One form of potential practical impact of your work is towards designers of support tools for decision making. Your research may help in making certain design choices. For example, would it make sense to let the AI explicitly play devil's advocate in certain situations, i.e., contrary to its own assessment, provide an explanation contrary to the clinician's own initial framing? Would it make sense to ask a clinician about their initial assessment and confidence before presenting the AI's second opinion and explanation? Perhaps you can think one step further about your results and conclusions towards design recommendations and add a paragraph in the discussion with some thoughts and recommendations directed towards such tool designers. | In the revised manuscript, we have added a new paragraph in the Discussion section (Section 5.2) with specific design recommendations for eliciting self-monitoring and supporting user reflection. We also build on the reviewer’s insightful suggestions (e.g., devil’s advocate role and pre-explanation confidence elicitation) and outline how these could be implemented or evaluated in future systems. We present the new added context below for your convenience:  Finally, systems could be designed to elicit users’ initial judgment and confidence before presenting AI recommendations—encouraging self-reflection and enabling tailored presentation of advice. This aligns with our finding that explanations are more effective when initial self-confidence is low. Additionally, tools could incorporate a “devil’s advocate” feature that presents counterfactual explanations challenging the user’s initial framing—even when the AI prediction aligns with the user’s intuition. Doing so could activate metacognitive conflict and help users re-evaluate their reasoning, similar to how human collaborators sometimes challenge each other’s assumptions. Furthermore, dynamically adapting explanation strength or framing based on user confidence and prediction confidence could support more calibrated reliance. We encourage designers to view explanations not just as static justifications, but as interactive mechanisms for cognitive support—capable of modulating trust, effort, and decision quality. |
| R3.6 | * Footnote 3: you say that you consider explanation efficiencies out of the scope of the paper. I would expect that a participant being confronted with a conflicting prediction is likely to study the reasoning in the explanation, checking it for being reasonable. So, isn't explanation efficiency inherently included in the scope of the paper? | We appreciate your thoughtful observation. Indeed, we agree that participants likely engage with explanations—especially when AI predictions conflict with their initial intuition—and that their perception of explanation quality can influence decision outcomes. In that sense, the effectiveness of explanations (i.e., how they support metacognitive processes) is central to our investigation. However, in Footnote 3, our intention was to distinguish between perceived effectiveness, which is implicitly captured through behavioral outcomes, and intrinsic explanation efficiency, meaning a technical assessment of how well the explanation mechanism captures the model’s true reasoning. Evaluating explanation mechanisms from a systems/algorithmic standpoint (e.g., comparing SHAP vs. LIME vs. feature attributions) was beyond the scope of this study. We have revised the footnote to better clarify this distinction between technical explanation fidelity and the behavioral effects of explanations on users. Below is the revised footnote:  Our assumption here is that the explanation mechanisms used in the study provide sufficiently interpretable and reasonable outputs to be meaningful to participants. We acknowledge that participants likely assess the plausibility of explanations, especially when AI predictions conflict with their own judgments. However, we do not evaluate the technical efficiency or fidelity of the explanation mechanism itself (i.e., how accurately the explanation reflects the internal reasoning of the model). Such system-level comparisons between explanation methods (e.g., SHAP, LIME, rule-based) are beyond the scope of this study, which focuses instead on the behavioral and metacognitive effects of explanations as perceived by human users. |
| R3.7 | * In 3.2.1 you refer to "two AI models". At that point in the paper, the reader doesn't know yet what two AI models you are referring to. A reader learns about it in 3.4.5. Perhaps better to explain the two AI models already in 3.2.1. | We thank you very much for this point. We have now added details of both models in section 3.2.2. |
| R3.8 | * In section 3.4.5 you present two models. It would be handy to present the accuracies of both models as measured with the labels of the golden standard (276 sentences with labels assigned by experts). | We thank you very much for this point. We have now added accuracies of both models in section 3.2.2. |
| R3.9 | * In the conclusion, you mention that there were cases where both AI and human expert were incorrect, but that the explanations triggered a self-monitoring process and led to correct decisions. It would be nice to have a few examples of these in the paper. | We appreciate this suggestion. We provide some examples of AI explanations of incorrect AI recommendations when participants arrive at the correct final judgment even when the initial framings of these tasks were incorrect in the conclusion section. These explanations that accompanied incorrect recommendations appear to be unrelated to the diagnosis. We present the new added context below for your convenience:  Our findings show that, contrary to Jussupow et al.’s [60] assumptions, at least in some cases, even though both AI and human agents are incorrect, explanations may be triggering self-monitoring processes and lead to correct decisions. For instance, when AI recommendations are incorrect, participants often arrive at the correct final judgment if those recommendations are accompanied by explanations that appear unrelated to the diagnosis (e.g., referencing terms like “internal auditory,” “canal,” “left,” or “cochlear nerve” as indicators of an abnormal diagnosis). This occurs even when the initial framings of these tasks were incorrect. |
